# Dietary Intake as an Undermeasured Potential Mediator: The Ethical Case for Complete and Transparent Dietary Reporting in GLP-1 Receptor Agonist Trials

**DOI:** 10.3390/nu18162722

**Published:** 2026-08-20

**Authors:** Jessica N. Strosahl, Thomas R. Ziegler, Rani H. Singh

**Affiliations:** 1 Department of Human Genetics, Emory University, Atlanta, GA 30322, USA; jstrosa@emory.edu; 2 Department of Medicine, Division of Endocrinology, Metabolism and Lipids, Emory University, Atlanta, GA 30322, USA; tzieg01@emory.edu

**Keywords:** glucagon-like peptide-1 receptor agonists, dietary intake assessment, registered dietitian nutritionists, research ethics, clinical trial reporting

## Abstract

Glucagon-like peptide-1 receptor agonists (GLP-1RAs) are transforming obesity management, yet landmark trials often incompletely measure and report a central mediator of response: dietary intake. Because GLP-1RAs promote weight loss primarily by suppressing appetite and reducing energy consumption, capturing changes in dietary intake is essential to interpreting efficacy, safety, and translation into clinical care. This perspective argues that failure to measure and report comprehensive dietary intake raises concerns under four core bioethical principles. It undermines autonomy by limiting evidence for shared decision-making across the treatment continuum. It challenges non-maleficence because inadequate energy, protein, and micronutrient intake may contribute to lean mass loss and nutrient deficiencies, particularly in vulnerable populations. It weakens beneficence because failing to report a measurable mediator diminishes the scientific value of research requiring substantial investment and participant burden. It raises justice concerns because the equitable distribution of assessment burden and access to resulting nutritional care cannot be evaluated without transparent reporting. To meet the ethical and scientific obligations of clinical research, future trials should preregister dietary intake as a key secondary or safety endpoint. Investigators should report total energy, macronutrient, and micronutrient distribution at baseline and serial follow-up alongside dietary assessment methods, food composition databases, and assessor training. Journal editors, funders, and regulatory bodies should support development of consensus-based, nutrition-specific reporting recommendations aligned with CONSORT principles and encourage preregistration of dietary trial endpoints. Registered dietitian nutritionists should be prospectively integrated into GLP-1RA trial teams from design through data interpretation. Advancing the standard of obesity care requires that the research community look beyond the scale and ground clinical translation in the nutritional context in which pharmacotherapy operates.

## 1. Introduction

The approval and rapid adoption of glucagon-like peptide-1 receptor agonists (GLP-1RAs) into clinical practice represents one of the most consequential pharmacological developments in obesity treatment in decades. Landmark trials including STEP 1 [1], SURMOUNT-1 [2], LEADER [3], and SCALE [4] have consistently demonstrated significant reductions in body weight and improvements in glycemic and cardiometabolic outcomes, generating widespread clinical enthusiasm and rapidly expanding indications. Yet a critical dimension of these trials remains incompletely measured and inconsistently reported: what participants actually ate during the studies. Recent analyses demonstrate that dietary intake is rarely assessed or reported in GLP-1RA trials, with only a small minority of studies including even limited nutritional measures [5,6]. This omission is particularly consequential given emerging evidence that GLP-1RA therapy results in significant reductions in lean mass (LM) [7,8]. Global health guidance, including the World Health Organization recommendations for integrated dietary and lifestyle intervention in GLP-1RA treatment models [9], reinforces the rationale for capturing these data within trials. While prior work has highlighted the scientific and translational limitations of missing dietary data in GLP-1RA trials [10], the ethical implications have not been examined. Here, we argue that incomplete measurement and failure to report dietary intake can undermine four core principles of research bioethics: autonomy, non-maleficence, beneficence, and justice [11].

## 2. Dietary Change Is a Critical, but Incompletely Measured, Mediator

GLP-1RAs produce weight loss through central and peripheral pathways that suppress appetite, slow gastric emptying, and enhance satiety signaling, thereby reducing energy intake [12]. Animal studies suggest additional effects beyond energy restriction, including preservation of resting energy expenditure [13] and activation of brown adipose tissue [14], though human evidence remains limited [15]. Nevertheless, reduced energy intake remains the major, though not exclusive, contributor to weight loss in humans. Its magnitude, timing, and nutrient composition are foundational to mechanistic interpretation and clinical translation.

In many pivotal obesity trials, participants in both drug and placebo arms receive identical behavioral counseling and a prescribed energy deficit, commonly 500 kcal/day. Without measuring actual dietary intake, investigators cannot determine whether between-group differences reflect pharmacological appetite suppression, differential adherence to the dietary prescription, or both. Failure to report a potential treatment mediator is inconsistent with current recommendations from the International Committee of Medical Journal Editors for complete, accurate, and non-misleading trial reporting and with the 2024 Declaration of Helsinki requirement for timely, complete, and accurate research reporting [16,17]. We contend that the ethical concern is strongest when the omitted information is foreseeable, feasible to collect, and necessary to interpret benefit or harm. This omission carries distinct ethical weight across four core principles of research bioethics: autonomy, non-maleficence, beneficence, and justice.

### 2.1. Autonomy

Autonomy, or respect for persons, concerns both valid consent and the quality of information available for subsequent health decisions [11]. The 2024 Declaration of Helsinki identifies free and informed consent as an essential component of respect for individual autonomy [16]. Incomplete dietary measurement does not by itself invalidate trial consent, because informed consent need not provide a complete mechanistic account of an intervention. The stronger ethical concern is that trials routinely prescribe an energy deficit yet fail to report whether and how intake changed. This leaves participants, future patients, and clinicians with inadequate evidence to understand what the medication enabled, which nutritional behaviors may require continued support, and how to plan for discontinuation. Published data show substantial weight regain after GLP-1RA discontinuation [18,19], whereas continued exercise and behavioral support may improve weight maintenance [20]. Transparent dietary reporting therefore supports shared decision-making, particularly regarding discontinuation planning and long-term weight maintenance.

### 2.2. Non-Maleficence

Non-maleficence requires that researchers assess, minimize, and monitor foreseeable risks and burdens [11,16]. Failure to measure and report dietary intake in GLP-1RA trials leaves a potentially modifiable mediator of nutritional risk unexamined. LM loss has emerged as a notable clinical concern based on recent meta-analyses of GLP-1RA trials [7,21]. While some LM loss is expected during any energy deficit [22,23,24], the degree to which observed losses reflect unavoidable pharmacology versus inadequate dietary protein intake and/or the absence of resistance training cannot be determined from current reports. Adequate dietary protein intake, reported in g/day or g/kg/day, together with progressive resistance training, is a potentially modifiable strategy for preserving LM during energy restriction [25,26], yet most studies report neither with sufficient granularity. Body composition outcomes should therefore be interpreted alongside dietary intake, physical activity, and functional measures rather than attributed automatically to an unavoidable drug effect. This gap has direct implications for older adults and others at increased risk of sarcopenia, functional decline, or malnutrition.

This concern extends beyond macronutrients. Substantial reductions in energy intake observed during GLP-1RA treatment, sometimes falling below 800 kcal/day in clinical practice [27], may increase the risk of micronutrient deficiencies, including vitamin B12, iron, calcium, folate, and zinc. Without dietary intake data, nutritional risks remain unquantifiable and therefore unaddressed in both trial interpretation and clinical guidance. Furthermore, improvements in HbA1c, triglycerides, and other cardiometabolic biomarkers [1,2,4] cannot be fully interpreted in relation to concurrent dietary change.

### 2.3. Beneficence

Beneficence requires that researchers maximize the scientific benefit and clinical utility of each trial [11,16]. The 2024 Declaration of Helsinki requires scientifically sound and rigorous research designed to produce reliable, valid, and valuable knowledge and avoid research waste [16]. This obligation extends beyond trial participants to the broader population that will be treated based on published findings. Participants accept burden and risk with the expectation that the knowledge generated will be scientifically interpretable and clinically useful. When a major, measurable mediator of response is omitted, a trial may still establish efficacy but yield less information about mechanism, heterogeneity of response, nutritional risk, and implementation. This avoidable loss of information diminishes the benefit derived from participants’ contributions. GLP-1RA trials span both publicly funded investigator-initiated and industry-sponsored studies; in both settings, the obligation to generate complete and interpretable findings remains constant regardless of funding source.

### 2.4. Justice

Justice concerns the fair distribution of research burdens and benefits [11]. The 2024 Declaration of Helsinki directs researchers to consider how benefits, risks, and burdens are distributed in the context of structural inequities [16]. Enhanced dietary assessment adds measurement burden to trial participation. Transparent reporting of assessment methods and participant demands is needed to determine whether this burden is distributed equitably across trial sites and participants, or falls disproportionately on those with greater logistical or literacy-related barriers to detailed dietary reporting. Justice also extends to the benefits of the knowledge generated: nutrition-related recommendations derived from GLP-1RA trials may have limited real-world applicability if access to registered dietitian nutritionist (RDN)-supported care is uneven across clinical settings. Transparent dietary reporting can therefore help clarify both who bears the burden of generating nutritional data and whether the resulting guidance can be implemented equitably in routine care.

## 3. A Path Forward

The scientific and ethical standards described here are achievable (Table 1) and directly responsive to the documented safety concerns and evidence gaps established above. Dietary assessment is feasible using validated approaches, although the intensity of assessment should be matched to the research question and balanced against participant burden, measurement error, and available resources. Timing of dietary assessment and any planned mediation analyses should be specified a priori. Future GLP-1RA trials should preregister dietary intake as a key secondary or safety endpoint, with reporting stratified by treatment arm at baseline and serial follow-up. Investigators should also disclose dietary assessment instruments and analysis methods, including nutrient analysis software, food composition databases, and personnel training, to support reproducibility and interpretation. Validated dietary assessment instruments, including multiple-day food records, 24-h recalls, food frequency questionnaires, and increasingly, technology-assisted dietary monitoring [28], should be selected based on the research question and applied with sufficient rigor to support mechanistic interpretation [29]. Reporting should capture not only total energy intake but also macronutrient and micronutrient composition and dietary supplements, with protein reported in clinically interpretable units such as g/day and g/kg/day. Physical activity and resistance-training prescriptions, adherence, and changes over time should also be reported alongside LM outcomes. Journal editors, peer reviewers, funders, regulatory bodies, and investigators should work toward consensus-based, nutrition-specific reporting standards aligned with CONSORT principles [30]. 

Systematic inclusion of RDNs in GLP-1RA trial teams is central to implementation. As the credentialed experts in dietary assessment methodology and medical nutrition therapy, RDNs are uniquely positioned to contribute to protocol development, dietary data collection, and interpretation of nutrition-related outcomes. While the specific role of the RDN should reflect study design, prospective integration is warranted for trials with dietary intake endpoints and ensures nutrition counseling, dietary assessment, and nutritional risk monitoring are treated as core trial infrastructure rather than ancillary procedures.

### Limitations

This perspective has limitations. As a narrative rather than systematic synthesis, it is subject to literature-selection bias, and no direct evidence yet demonstrates that improved dietary reporting translates into better patient outcomes. Self-reported dietary intake, the most feasible assessment method in large multicenter trials, carries known systematic measurement error such as under- and over-reporting and may not capture true intake even when validated instruments are used [31,32]. The recommendations proposed here also entail meaningful practical costs, including increased participant burden, additional personnel and training requirements, and resource demands that may be difficult to implement uniformly across all trial centers and healthcare systems internationally. Finally, we do not intend for these standards to be adopted unilaterally; formal multidisciplinary consensus development involving investigators, RDNs, journal editors, patient representatives, and regulatory bodies should precede adoption of mandatory reporting requirements.

## 4. Conclusions

GLP-1RAs reduce body weight and improve metabolic outcomes in large part by decreasing energy intake, and that is precisely why dietary intake must be measured and reported. Persistent undermeasurement and incomplete reporting of these data are not minor methodological oversights; they represent an ethical and scientific gap compromising autonomy, non-maleficence, beneficence, and justice. As multi-agonist anti-obesity drugs move to the forefront of public and scientific interest, pharmacology and nutrition should not be treated as independent domains when treatment effects occur in part through changes in eating behavior. The research community, journal editors, funders, and regulatory bodies share responsibility for ensuring complete and transparent dietary reporting in clinical trials. Patients, clinicians, and policymakers deserve evidence that reflects what occurred during treatment—including what participants ate.

## Figures and Tables

**Table 1:** Minimum standards for dietary intake assessment and reporting in GLP-1RA clinical trials.

Domain	Recommendation	Rationale	Responsible Party
Trial registration	Preregister dietary intake as a key secondary or safety endpoint and specify a priori for mediation analysis.	Enforces transparent collection of the primary mediator, reduces selective reporting, and captures dietary change.	Investigators, regulatory bodies, funders
Trial design	Prospectively integrate registered dietitian nutritionists into the clinical research team; role (core team member or consultant) should be defined by study design.	Ensures expertise in dietary assessment methodology, medical nutrition therapy, and interpretation of nutrition-related outcomes.	Investigators
	Utilize a validated dietary intake assessment appropriate for the research question.	Improves methodological rigor and reproducibility.	Investigators
	Measure dietary intake at baseline and serial follow-up across all study arms.	Enables assessment of adherence, temporal dietary change, and attribution of treatment effects.	Investigators
Publication	Report details of dietary intake assessment and analysis, including nutrient analysis software, food composition database, and assessor training/credentials.	Supports transparency, reproducibility, and interpretation of dietary data quality.	Investigators
	Support development and adoption of consensus-based, nutrition-specific reporting standards aligned with CONSORT principles.	Promotes nutritional rigor, reproducibility, and standardized reporting while allowing formal consensus development before mandatory requirements are adopted.	Journal editors, peer reviewers, funders, regulatory bodies, investigators
	Report comprehensive dietary intake data, including total energy, macronutrients, micronutrients, and dietary supplements.	Equips researchers and clinicians with the dietary context to interpret study findings.	Investigators
	Report dietary intake results stratified by treatment arm and over time.	Facilitates the mechanistic interpretation of treatment effects and comparison between intervention and control groups.	Investigators
	Report physical activity and resistance-training prescriptions, adherence, and changes over time alongside lean mass outcomes.	Helps distinguish potentially modifiable lean mass loss from drug effect and supports safer clinical translation.	Investigators

## Data Availability

No new data were created or analyzed in this study. Data sharing is not applicable to this article.

## Abbreviations

The following abbreviations are used in this manuscript:

GLP-1RAs	Glucagon-like peptide-1 receptor agonists
LM	Lean mass
RDN	Registered dietitian nutritionist

## References

[1] Wilding J.P.H., Batterham R.L., Calanna S., Davies M., Gaal L.F.V., Lingvay I., McGowan B.M., Rosenstock J., Tran M.T.D., Wadden T.A. (2021). Once-Weekly Semaglutide in Adults with Overweight or Obesity. N. Engl. J. Med..

[2] Jastreboff A.M., Aronne L.J., Ahmad N.N., Wharton S., Connery L., Alves B., Kiyosue A., Zhang S., Liu B., Bunck M.C. (2022). Tirzepatide Once Weekly for the Treatment of Obesity. N. Engl. J. Med..

[3] Marso S.P., Daniels G.H., Brown-Frandsen K., Kristensen P., Mann J.F.E., Nauck M.A., Nissen S.E., Pocock S., Poulter N.R., Ravn L.S. (2016). Liraglutide and Cardiovascular Outcomes in Type 2 Diabetes. N. Engl. J. Med..

[4] Pi-Sunyer X., Astrup A., Fujioka K., Greenway F., Halpern A., Krempf M., Lau D.C.W., Roux C.W.l., Ortiz R.V., Jensen C.B. (2015). A Randomized, Controlled Trial of 3.0 mg of Liraglutide in Weight Management. N. Engl. J. Med..

[5] Jansson A.K., Gómez-Martín M., Hedin L., Clarke E.D., Cross V., Stanford J., Taylor R.M., Bogl L.H., De Vlieger N., Koochek A. (2026). A Systematic Review Identifying Critical Evidence Gaps in Reporting Dietary Change in Randomized Controlled Trials Prescribing Liraglutide, Semaglutide, or Tirzepatide. Obes. Rev..

[6] Babazadeh D., Wyatt S., Steinberg F.M. (2025). Examining the Omission of Dietary Quality Data in Glucagon-Like Peptide 1 Clinical Trials: A Scoping Review. Adv. Nutr..

[7] Karakasis P., Patoulias D., Fragakis N., Mantzoros C.S. (2025). Effect of glucagon-like peptide-1 receptor agonists and co-agonists on body composition: Systematic review and network meta-analysis. Metabolism.

[8] Neeland I.J., Linge J., Birkenfeld A.L. (2024). Changes in lean body mass with glucagon-like peptide-1-based therapies and mitigation strategies. Diabetes Obes. Metab..

[9] Celletti F., Farrar J., De Regil L. (2026). World Health Organization Guideline on the Use and Indications of Glucagon-Like Peptide-1 Therapies for the Treatment of Obesity in Adults. JAMA.

[10] Dashti H.S., Szczerbinski L. (2025). The Silent Weight of Nutrition Data in Glucagon-Like Peptide-1 Receptor Agonists Clinical Trials. Adv. Nutr..

[11] Beauchamp T.L., Childress J.F. (1979). Principles of Biomedical Ethics.

[12] Drucker D.J. (2022). GLP-1 physiology informs the pharmacotherapy of obesity. Mol. Metab..

[13] Bredum S.K., Jacobsen J.M., Halling J.F., Blom I., Lewis C.T.A., Ochala J., Fredholm M., Lundh S., Schmücker M., Ozenne B. (2026). Semaglutide mitigates the loss of fat-free mass and decreased energy expenditure observed after diet restriction. Insights from an obese minipig model. Am. J. Physiol. Endocrinol. Metab..

[14] Ma T., Song F., Pan Y., He Y., Cao X., Zhang Y., Song G., Ren L. (2025). Distinct effects of semaglutide and tirzepatide on metabolic and inflammatory gene expression in brown adipose tissue of mice fed a high-fat, high-fructose diet. Front. Nutr..

[15] Hropot T., Herman R., Janez A., Lezaic L., Jensterle M. (2023). Brown Adipose Tissue: A New Potential Target for Glucagon-like Peptide 1 Receptor Agonists in the Treatment of Obesity. Int. J. Mol. Sci..

[16] World Medical Association (2025). World Medical Association Declaration of Helsinki: Ethical Principles for Medical Research Involving Human Participants. JAMA.

[17] International Committee of Medical Journal Editors Recommendations for the Conduct, Reporting, Editing, and Publication of Scholarly Work in Medical Journals. https://www.icmje.org/recommendations/.

[18] Berg S., Stickle H., Rose S.J., Nemec E.C. (2025). Discontinuing glucagon-like peptide-1 receptor agonists and body habitus: A systematic review and meta-analysis. Obes. Rev..

[19] West S., Scragg J., Aveyard P., Oke J.L., Willis L., Haffner S.J.P., Knight H., Wang D., Morrow S., Heath L. (2026). Weight regain after cessation of medication for weight management: Systematic review and meta-analysis. BMJ.

[20] Jensen S.B.K., Blond M.B., Sandsdal R.M., Olsen L.M., Juhl C.R., Lundgren J.R., Janus C., Stallknecht B.M., Holst J.J., Madsbad S. (2024). Healthy weight loss maintenance with exercise, GLP-1 receptor agonist, or both combined followed by one year without treatment: A post-treatment analysis of a randomised placebo-controlled trial. EClinicalMedicine.

[21] Jiao R., Lin C., Cai X., Wang J., Wang Y., Lv F., Yang W., Ji L. (2025). Characterizing body composition modifying effects of a glucagon-like peptide 1 receptor-based agonist: A meta-analysis. Diabetes Obes. Metab..

[22] Chaston T.B., Dixon J., O’Brien P.E. (2007). Changes in fat-free mass during significant weight loss: A systematic review. Int. J. Obes..

[23] Huang J., Li Y., Chen M., Cai Z., Cai Z., Jiang Z. (2024). Comparing caloric restriction regimens for effective weight management in adults: A systematic review and network meta-analysis. Int. J. Behav. Nutr. Phys. Act..

[24] Tinsley G.M., Heymsfield S.B. (2024). Fundamental Body Composition Principles Provide Context for Fat-Free and Skeletal Muscle Loss with GLP-1 RA Treatments. J. Endocr. Soc..

[25] Cigrovski Berkovic M., Ruzic L., Cigrovski V., Strollo F. (2025). Saving muscle while losing weight: A vital strategy for sustainable results while on glucagon-like peptide-1 related drugs. World J. Diabetes.

[26] Mechanick J.I., Butsch W.S., Christensen S.M., Hamdy O., Li Z., Prado C.M., Heymsfield S.B. (2025). Strategies for minimizing muscle loss during use of incretin-mimetic drugs for treatment of obesity. Obes. Rev..

[27] Al-Najim W., Raposo A., BinMowyna M.N., le Roux C.W. (2025). Unintended Consequences of Obesity Pharmacotherapy: A Nutritional Approach to Ensuring Better Patient Outcomes. Nutrients.

[28] Chotwanvirat P., Prachansuwan A., Sridonpai P., Kriengsinyos W. (2024). Advancements in Using AI for Dietary Assessment Based on Food Images: Scoping Review. J. Med. Internet Res..

[29] Bailey R.L. (2021). Overview of dietary assessment methods for measuring intakes of foods, beverages, and dietary supplements in research studies. Curr. Opin. Biotechnol..

[30] Rigutto-Farebrother J., Ahles S., Cade J., Murphy K.J., Plat J., Schwingshackl L., Roche H.M., Shyam S., Lachat C., Minihane A.-M. (2023). Perspectives on the application of CONSORT guidelines to randomised controlled trials in nutrition. Eur. J. Nutr..

[31] Bajunaid R., Niu C., Hambly C., Liu Z., Yamada Y., Aleman-Mateo H., Anderson L.J., Arab L., Baddou I., Bandini L. (2025). Predictive equation derived from 6,497 doubly labelled water measurements enables the detection of erroneous self-reported energy intake. Nat. Food.

[32] Kirkpatrick S.I., Troiano R.P., Barrett B., Cunningham C., Subar A.F., Park Y., Bowles H.R., Freedman L.S., Kipnis V., Rimm E.B. (2022). Measurement Error Affecting Web- and Paper-Based Dietary Assessment Instruments: Insights From the Multi-Cohort Eating and Activity Study for Understanding Reporting Error. Am. J. Epidemiol..

